# Two New Daucane Sesquiterpenoids from *Daphne aurantiaca*

**DOI:** 10.3390/molecules170910046

**Published:** 2012-08-24

**Authors:** You-Xing Zhao, Sheng-Zhuo Huang, Qing-Yun Ma, Wen-Li Mei, Hao-Fu Dai

**Affiliations:** Institute of Tropical Bioscience and Biotechnology, Chinese Academy of Tropical Agricultural Sciences, Haikou 571101, Hainan, China; Email: huangshengzhuo@yahoo.com.cn (S.-Z.H.); maqy1024@163.com (Q.-Y.M.); meiwenli@yahoo.com.cn (W.-L.M.)

**Keywords:** *Daphne aurantiaca*, Thymelaeceae, daucane

## Abstract

Two new daucane sesquiterpenoids 1β,2β-epoxy-10(*H*)α-dauca-11(12)-ene-7α,14-diol (**1**) and 1α,2α-epoxy-10(*H*)α-dauca-11(12)-ene-7α,14-diol (**2**) were isolated from the plateau medicinal plant *Daphne aurantiaca* Diels. (Thymelaeceae). Their structures were elucidated by 1D and 2D NMR spectroscopy, as well as HR-ESI-MS data.

## 1. Introduction

*Daphne aurantiaca* Diels. (Thymelaeceae) is an evergreen shrub widely distributed in the plateau area of southwest China and used traditionally in folk medicine [1]. The bark is used for the treatment of tranumatic injury [2]. The Thymelaeceae plants were claimed to provide good medicines against tumors [3], inflammation [2], antihyperglycemic [4], and neurotrophic [5]. Recently, some phenols and daphane diterpenoids from *Daphne acutiloba* have showed strong anti-HIV-1 activities [6,7]. Previous studies have also reported a series of sesquiterpenoids and diterpenoids isolated from *D. aurantiaca* with potential anti-inflammatory activities [2]. In order to study new bioactive constituents from this plant, the phytochemical investigation have been carried out, and two new dacane sesquiterpenoids named 1β,2β-epoxy-10(*H*)α-dauca-11(12)-ene-7α,14-diol (**1**) and 1α,2α-epoxy-10(*H*)-α-dauca-11(12)-ene-7α,14-diol (**2**) were isolated. Herein, we describe the isolation, structural elucidation of the new compounds as well as their anti-HIV-1 and acetylcholinesterase (AChE) inhibitory activities.

## 2. Results and Discussion

Compound **1** was obtained as a yellow oil, and its molecular formula was assigned as C_15_H_24_O_3_ by HR-ESI-MS (*m/z* 275.1628 [M+Na]^+^ calcd. for C_15_H_24_O_3_Na 275.1623) and NMR data (Table 1), indicating four degrees of unsaturation. 

**Table 1:** NMR data of compounds **1** and **2** in CDCl_3_ (^1^H: 500 MHz; ^13^C: 125 MHz; *δ* in ppm, *J* in Hz).

Position	1		2	
	*δ*_H_	*δ*_C_	*δ*_H_	*δ*_C_
**1**	-	67.5	-	67.5
**2**	3.27 (1 H, dd, 6.8, 7.7)	59.4	3.15 (dd, 1 H, 1.6, 4.8)	61.8
**3**	2.16 (1 H, ddd, 3.1, 7.7, 14.2)	27.0	2.15 (ddd, 1 H, 3.1, 4.8, 15.6)	26.8
	1.53 (1 H, ddd, 6.8, 13.3, 14.2)		1.53 (ddd, 1 H, 1.6, 13.3, 15.6)	
**4**	1.88 (1 H, m)	48.3	2.25 (ddd, 1 H, 3.1, 13.3, 13.5)	46.1
**5**	-	43.3	-	41.8
**6**	1.85 (1 H, dd, 12.6, 16.3)	50.1	1.87 (1 H,dd, 5.0, 16.3)	48.4
	1.66 (1 H, dd, 1.6, 16.3)		1.46 (1 H, dd, 11.6, 16.3)	
**7**	3.85 (1 H,dd, 1.6, 12.6)	72.6	4.29 (1 H,dd, 5.0, 11.6)	68.2
**8**	1.61 (1 H, m)	42.9	1.47 (1 H, m)	43.3
	1.41 (1 H, m)		1.40 (1 H, m)	
**9**	1.92 (1 H, m)	29.9	1.85 (1 H, m)	29.6
	1.77 (1 H, m)		1.76 (1 H, m)	
**10**	2.95 (1 H, ddd, 2.2, 13.5, 13.6)	50.0	2.92 (1 H, ddd, 2.2, 13.4, 14.3)	50.7
**11**	-	149.0	-	148.3
**12**	4.79 (1 H, d, 1.3)	113.4	4.85 (1 H, d, 1.3)	113.9
	4.68 (1 H, d, 1.3)		4.74 (1 H, d, 1.3)	
**13**	1.66 (3 H, s)	23.2	1.73 (3 H, s)	23.4
**14**	3.90 (1 H, d, 12.9)	60.6	4.07 (1 H, d, 12.5)	61.8
	3.75 (1 H, d, 12.9)		3.30 (1 H, d, 12.5)	
**15**	0.99 (3 H, s)	18.3	0.85 (3 H, s)	18.3

**Figure 1:**
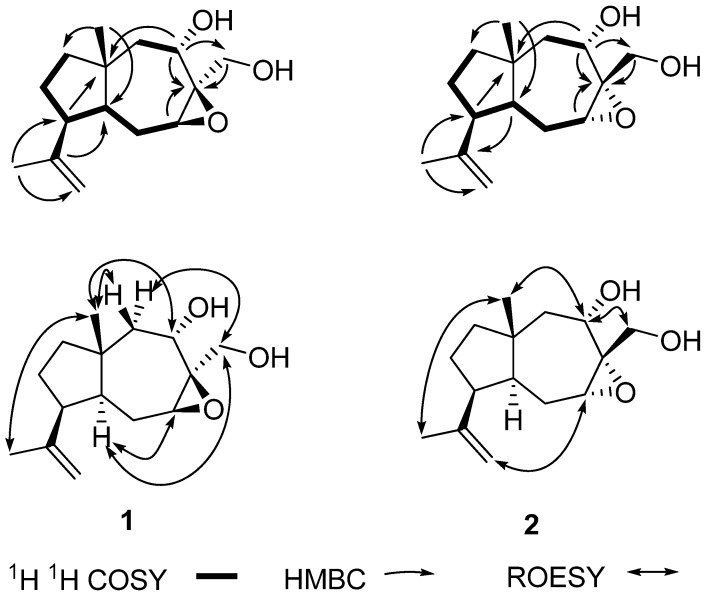
Key 2D NMR correlations of compounds **1** and **2**.

The IR spectrum displayed the presence of hydroxyls (3,425 cm^−1^) and olefinic bond (1,635 cm^−1^) absorptions. Analysis of its ^13^C-NMR and DEPT spectra (Table 1) showed the presence of 15 carbon resonances. These carbons were assigned to two singlet methyl groups, six methylenes (including one oxygenated and one terminal double bond), four methines (including two oxygenated), and three quaternary carbons (including one oxygenated). Comparing the ^13^C-NMR data (Table 1) of **1** with those of dauca-1,11-diene-7α,14-diol [2] showed that **1** had the same daucane skeleton. The differences were that the C-1 (*δ*_C_ 127.4) and C-2 (*δ*_C_ 143.8) in dauca-1,11-diene-7α,14-diol shifted up-field to *δ*_C_ 67.5 and 59.4 in compound **1**, respectively, corresponding to H-2 [*δ*_H_ 5.78 (1 H, d, *J* = 5.6 Hz)] in dauca-1,11-diene-7*α*,14-diol shifted up-field to *δ*_H_ 3.27 (1 H, dd, *J* = 6.8, 7.7 Hz) in **1**, which suggested that compound **1** was generated from dauca-1,11-diene-7α,14-diol with C-1-C-2 double bond oxidized to a epoxy group, combined with the analysis of its molecular formula. The key HMBC correlations (Figure 1) of **1** from H-2 [*δ*_H_ 3.27 (1 H, dd, *J* = 6.8, 7.7 Hz)], H-7 [*δ*_H_ 3.85 (1 H, d, *J* = 1.5, 12.6 Hz)], and H-14 to C-1 [*δ*_C_ 67.5 (s)] further clarified this hypothesis.

**Figure 2:**
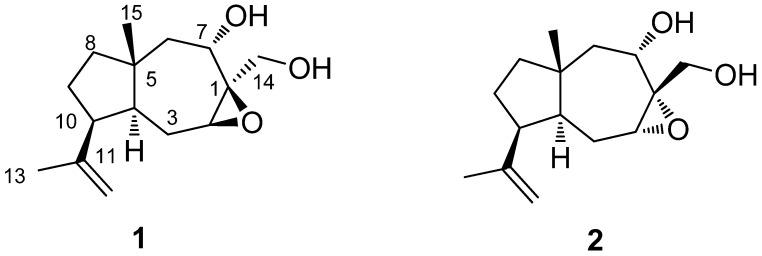
Structures of compounds **1** and **2**.

The other correlations in the HMBC and ^1^H-^1^H COSY (Figure 1) spectrum further confirmed the atom connectivity in **1**. The relative configurations of the stereogenic centers (C-4, C-5, and C-7) of **1** were elucidated by comparison of NMR data with dauca-1,11-diene-7α,14-diol and NOESY spectrum (Figure 1) and determined to be the same as those of dauca-1,11-diene-7α,14-diol with β-orientations of CH_3_-15 and H-7 and α-orientation of H-4. The β-orientation of the epoxy group was elucidated by NOE of H-2/H-14 [*δ*_H_ 3.90 (1 H, d, *J* = 12.9 Hz)] and H-2/H-4 [*δ*_H_ 1.88 (1 H, m)]. Moreover, the α-orientation of H-10 in compound **1** was determined by NOE of H-13 [*δ*_H_ 1.66 (3 H, s, H-13)]/H-15 [*δ*_H_ 0.99 (3 H, s, H-15)]. Thus, the structure of compound **1** was assigned as showed in Figure 2, and this compound was named 1β,2β-epoxy-10(*H*)α-dauca-11(12)-ene-7α,14-diol.

Compound **2** was obtained as a yellow oil and had the same molecular formula as **1** based on HR-ESI-MS *m/z* [M+Na]^+^ 275.1617 (calcd for C_15_H_24_O_3_Na, 275.1623), with four degrees of unsaturation. The IR spectrum also displayed the presence of hydroxyls (3,419 cm^−1^) and olefinic bond (1,636 cm^−1^) absorptions. The ^1^H and ^13^C-NMR spectroscopic data of **2** was extremely similar to those of **1** except that C-2 (*δ*_C_ 59.4) in compound **1**shifted down-field to *δ*_C_ 61.8 in **2** and H-2 (*δ*_H_ 3.27) in **1** shifted up-field to *δ*_H_ 3.15 in **2**, which hinted compound **2** was the isomer of **1** with different configuration of C-1 and C-2 (epoxy group). This was confirmed by the NOESY experiment. The relative configurations of the stereogenic centers (C-4, C-5, C-7, and C-10) of **2** were determined to be the same as those of compound **1** by NOE of H-15 (*δ*_H_ 0.85)/H-7 (*δ*_H_ 4.29) and H-15/H-13 (*δ*_H_ 1.73). The *a*-orientation of epoxy group was deduced by key NOE of H-2/H-12 (*δ*_H_ 4.85) and H-7/H-14 (*δ*_H_ 4.07 and 3.30). The correlations in the HMBC and ^1^H-^1^H COSY spectrum (Figure 1) further confirmed the assignments of **2**. Thus, the structure of compound **2** was assigned as showed in Figure 2, and was named 1α,2α-epoxy-10(*H*)α-dauca-11(12)-ene-7,14-diol. Finally the anti-HIV-1 and inhibitory AChE activities of compounds **1** and **2** were evaluated. The two new compounds showed no activity against HIV-1 and AChE.

## 3. Experimental

### 3.1. General

Spectra were recorded on the following instruments: optical rotation, Jasco P-1020 polarimeter; UV, Shimadzu double-beam 210A spectrometer; IR, Tensor 27 spectrometer (KBr pellets); NMR; Bruker AV-400 or a DRX-500 spectrometer (TMS as an internal standard using CDCl_3_ as solvent); ESI-MS and HR-ESI-MS, API QSTAR Pulsar 1 spectrometer. Silica gel (200–300 mesh, Qingdao Marine Chemical Inc., Qingdao, China), RP-18 (40–70 μm, Fuji Silysia Chemical Ltd., Aichi, Japan) and Sephadex LH-20 (Amersham Biosciences, Uppsala, Sweden) were used for column chromatography. Semipreparative HPLC was performed on an Agilent 1100 liquid chromatograph equipped with a Zorbax SB-C_18_, 9.4 mm × 25 cm column. Fractions were monitored by TLC and spots were visualized by heating after spraying with 5% H_2_SO_4_ in ethanol.

### 3.2. Plant Material

The stems of *Daphne aurantiaca* Diels. were collected in Xianggelila Yunnan Province, People’s Republic of China. A voucher specimen (CHRX0001), identified by Prof. H. Sun and Dr. L.L. Yue (Kunming Institute of Botany, Chinese Academy of Sciences), was deposited in Institute of Tropical Bioscience and Biotechnology, Chinese Academy of Tropical Agricultural Sciences.

### 3.3. Extraction and the Isolation

The dried and powdered stems of *Daphne aurantiaca* (4.5 kg) were extracted with 95% EtOH (15 L) under reflux three times. The extract was concentrated and suspended in water followed by successive partition with petroleum ether (3 × 4 L) and EtOAc (3 × 4 L). The EtOAc extract (300 g) was separated by silica gel column using a gradient solvent CHCl_3_/MeOH (9:1–3:1 10 L) to afford fractions A–C. Fraction A (40 g) was separated by silica gel column using a gradient solvent petroleum ether/EtOAc (20:1–1:2 8 L) to afford fractions A1–A4. Fr. A2 (5g) was purified over Sephadex LH-20 column (CHCl_3_/MeOH 1:1 3 L) to give two subfractions A2-1 and A2-2. Subfr.A2-1 (2 g) was subjected to repeated RP-18 column (MeOH/H_2_O 2:1 2 L) to obtain the mixture two compounds **1** and **2**. The mixture (42.3 mg) was further separated by semi-preparative HPLC (MeOH/H_2_O 45:55 0.5 L) to yield **1** (11.7 mg) and **2** (8.5 mg).

*1β,2β-Epoxy-10(H)α-dauca-11(12)-ene-7,14-diol* (**1**). Yellow oil; [α]_*D*_^17^: +1.29 (*c* 0.145, MeOH); UV (MeOH) *λ*_max_ (logε) 202 (3.54), 229 (2.66); IR (KBr) *ν*_max_ 3425, 2950, 2878, 2858, 1635, 1454, 1383, 1107, 1067, 1055, 1024, 889; ^1^H and ^13^C-NMR see Table 1; ESI-MS positive *m/z* [M+Na]^+^ 275 (100); HR-ESI-MS *m/z* [M+Na]^+^ 275.1628 (calcd for C_15_H_24_O_3_Na, 275.1623).

*1α,2α-Epoxy-10(H)α-dauca-11(12)-ene-7,14-diol* (**2**). Yellow oil; C_15_H_2_4O3, [α]_*D*_^18^: +15.67 (*c* 0.305, MeOH); UV (MeOH) *λ*_max_ (logε) 202 (3.57), 237 (2.47); IR (KBr) *ν*_max _3419, 2949, 2930, 2855, 1636, 1451, 1382, 1024, 972, 890; ^1^H and ^13^C-NMR see Table 1; ESI-MS positive *m/z* [M+Na]^+^ 275 (100); HR-ESI-MS *m/z* [M+Na]^+^ 275.1617 (calcd for C_15_H_24_O_3_Na, 275.1623).

### 3.4. Anti-HIV-1 Bioassay

The anti-HIV activity was evaluated by the inhibition assay for the cytopathic effects of HIV-1 (EC_50_) and cytotoxicity assay against C8166 cell line (IC_50_) using MTT methods as described in the literature [8]. AZT (3'-azido-3'-deoxythymidine, Sigma-Aldrich 99%, Seelze, Germany) was used as positive control. The concentration of the antiviral sample reducing HIV-1 replication by 50% (EC_50_) was determined from the dose response curve and calculated by Reed and Muench method [9].

### 3.5. Bioassay of AChE Inhibitory Activity

Acetylcholinesterase inhibitory activity was assayed by the spectrophotometric method developed by Ellman [10] with slightly modification. *S-*Acetylthiocholine iodide, 5,5'-dithio-bis-(2-nitrobenzoic) acid (DTNB, Ellman’s reagent), acetylcholinesterase derived from human erythrocytes were purchased from Sigma Chemical (Perth, Australia). Compounds were dissolved in DMSO. The reaction mixture (totally 200 μL) containing phosphate buffer (pH 8.0), test compound (50 μM), and acetyl cholinesterase (0.02 U/mL), was incubated for 20 min (30 °C). The reaction was initiated by the addition of 20 μL of DTNB (0.625 mM) and 20 μL acetylthiocholine iodide (0.625 mM) for AChE inhibitory activity assay. The hydrolysis of acetylthiocholine was monitored at 405 nm after 30 min. Tacrine (Sigma-Aldrich 99%) was used as positive control with final concentration of 0.333 μM, and DMSO was used as negative control with final concentration of 0.1%. All the reactions were performed in triplicate. The percentage inhibition was calculated as follows: % inhibition = (E − S)/E × 100 (E is the activity of the enzyme without test compound and S is the activity of enzyme with test compounds).

## 4. Conclusions

The phytochemical investigation of *Daphne aurantiaca* led to the isolation of two new dacane sesquiterpenoids named 1β,2β-epoxy-10(H)α-dauca-11(12)-ene-7α,14-diol (**1**) and 1α,2α-epoxy-10(*H*)α-dauca-11(12)-ene-7α,14-diol (**2**), which were isomers. The evaluation on anti-HIV-1 and inhibitory AChE activities of two new dacane sesquiterpenoids showed these two isolates exhibited no activity against HIV-1 and AChE.
